# Changes in the Dentition of Small Dogs up to 4 Months of Age

**DOI:** 10.3390/ani12111417

**Published:** 2022-05-31

**Authors:** Gábor Lorászkó, Bence Rácz, László Ózsvári

**Affiliations:** 1 Department of Anatomy and Histology, University of Veterinary Medicine, H-1078 Budapest, Hungary; loraszko.gabor@univet.hu (G.L.); racz.bence@univet.hu (B.R.); 2 Department of Veterinary Forensics and Economics, University of Veterinary Medicine, H-1078 Budapest, Hungary

**Keywords:** age determination, deciduous tooth, dog trade, animal protection, animal welfare

## Abstract

**Simple Summary:**

National and EU legislation impose age restrictions for the rabies vaccination which is required in the export of dogs. This makes it important to know whether a particular dog is older than three months or not. In veterinary practice, age estimation is mostly based on dentition, although there is no standardized method described in the literature for determining the age of dogs under four months old and we found considerable variation in the references. We observed and recorded the changes in cranial shape and dentition of two Yorkshire Terriers born by caesarean section on 2 March 2018 up to four months of age. At the age of three months, both individuals showed the same characteristics of a wide gap between the upper maxillary incisors (i2 and i3) and the lower maxillary incisor and canine (i3 and c).

**Abstract:**

It is common practice in EU member states to permit the entry of dogs vaccinated against rabies at the age of at least 3 months. In the absence of easily applicable comparative data, subjective disputes emerge around age. The aim of our study was to observe the development of dog teeth. During birth, an abnormally lying Yorkshire Terrier fetus was stuck in the birth canal, which led to a caesarean section, hence, the exact date of birth was known. For the next 4 months, two puppies were examined weekly, and they showed the same development. The dogs were born without teeth. At the age of 4.5 weeks, the canines I appeared, together with the adjacent incisors (i3), and the second incisor (i2) also erupted at the age of 6 weeks. A week later, a first incisor (i1) also appeared. From the age of 2.5 months, the distance between the teeth was increasing, especially on the upper dental arch. At 3.5 months of age, only the bottom front incisors (i1) had not grown in a row, and the significant distance between the top incisors, comparable to the width of the tooth, was striking. Since only two dogs of one breed were involved in this case study, the observations cannot be generalized.

## 1. Introduction

It is estimated that there are 74.4 million dogs in the EU member states [1] and of those, 46,000 dogs are traded every month, with an annual turnover of EUR 1.3 billion, directly supporting the incomes of 300,000 people [2]. The link between dog trafficking and crime [3] is regularly addressed by the European Union [4,5] and national authorities [6,7], NGOs [8,9] and the media [10,11,12,13], primarily from an animal welfare perspective.

Traditionally, one of the most popular transport destinations from Hungary is Italy, where the relevant legislation is the imprisonment of up to 1 to 3 months and fines of EUR 3000–15,000 for importing animals without the appropriate documentation, which increases if the animal is less than 12 weeks old [14], while the documents accompanying the animals often contain false information [15]. Although the annual incidence of rabies in pets in EU member states has recently fallen to just a few cases, in neighboring Ukraine, the incidence of rabies is two orders of magnitude higher [16]. This underlines the importance of vaccination against rabies, and the associated minimum age for eligibility makes it important to estimate the age of puppies as accurately as possible. 

In the past, it was recommended to look at the dentition [17,18] of young and old dogs to distinguish them from each other, and the dentition was also considered the best way to estimate the age of a horse [19] when it was purchased, although its empirical system was described thousands of years later [20,21,22,23].

The timeline of tooth development was also used to identify the age of children employed in industrial production [24], a method that is still, after nearly two hundred years, scientifically accepted [25]. After human examinations, medical researchers studying the development of dentition in animals [26] compared the processes in animal species [27], including dogs, and found that nutrient and vitamin supply also have an effect on it [28]. More recent studies identified a wider range of influencing factors that are difficult to separate [29]. Rodents have proved to be the best model animal because of their ever-growing incisors [30].

In veterinary medicine, the changes in horse dentition with age has always been an important piece of knowledge [31] and communications are still issued on age determination based on teeth [32]. This procedure is still used on laboratory animals [33] and wild animals [34], such as fur-bearing animals [35] and wild boar [36].

In the modern era, the first choice for estimating the age of a dog was also its dentition [37]. The role of photographs, which are of a quality that can also be evaluated today, was later taken over by drawings [38]. The names of the teeth [39], their positioning [40], their approximation [41] and their exact structure [42] are described in detail in anatomy textbooks.

X-ray diagnostics may be used to determine the age of dogs on the basis of the appearance of certain tendons [43,44,45,46] and the progression of ossification [47,48,49,50,51] and changes in the positioning and structure of certain teeth [52,53]. The age of the dog can be determined by histopathological examination of the cerebellum up to 75 days of age [54] and ophthalmological examination can be used to determine the age of adult animals [55,56]. The age of a dog can only be determined to a limited extent [57,58,59,60], or not at all [61], on the basis of specific behaviors (walking, vocalization) [62] appearing from a few weeks of age, EEG examination [63] or neurological examination [64].

In practice, it would be advantageous if age could be recognized without special tests in order to determine whether the dog in question, on the basis of its age, could comply with the border crossing rules or whether the accompanying documents are authentic. There can be substantial variation in the rate of tooth eruption [65,66] but, for example, in the United States, no entry is allowed for any dog under eight weeks old [67] and, for commercial or adoption purposes, only dogs over six months of age are allowed [68], and they consider this determinable on the basis of the teeth [69]. The literature on the subject typically includes data on large and medium-sized dogs, for example, the dentition of 89 medium and large dogs [70] or 107 beagle puppies [71] were recorded; however, Roccaro [65] also studied the dentition of small dog breeds (eight Pomeranian dwarf spitz and two dwarf poodles).

In this case study, we followed the development of canine milk teeth from a known date of birth to 3.5 months of age in two Yorkshire Terriers. The long-term aim was to start collecting data that would be readily comparable retrospectively, and later, on the basis of a sufficient number of samples, to answer the question as to whether, in daily practice, age estimation based on dentition could possibly give acceptable accuracy within a certain breed.

## 2. Materials and Methods

After a caesarean section performed on a Biewer Yorkshire Terrier (the Yorkshire Terrier is not a separate breed due to a recessive gene defect in the white color), whose whelping was arrested due to lying abnormally on 2 March 2018, the owner presented the two surviving puppies for examination on day 10, 18, 25, 32, 41, 46, 53, 62, 69, 81 and 102 of life. The photos were taken by the following camera and accessories: Canon EOS 600D 1/200 s, ISO 400, Canon EF-S 60 mm f/2.8 and Canon Speedlite 430 EX II EOS.

We used similar marking on the images:

- Age in days and rounded in weeks;
- Dentition characteristics: missing tooth is marked in white, erupting tooth in red, growing tooth in yellow and deciduous tooth in line in green square;
- Graphical representation of the time between birth and four months of age.

**Figure 1:**
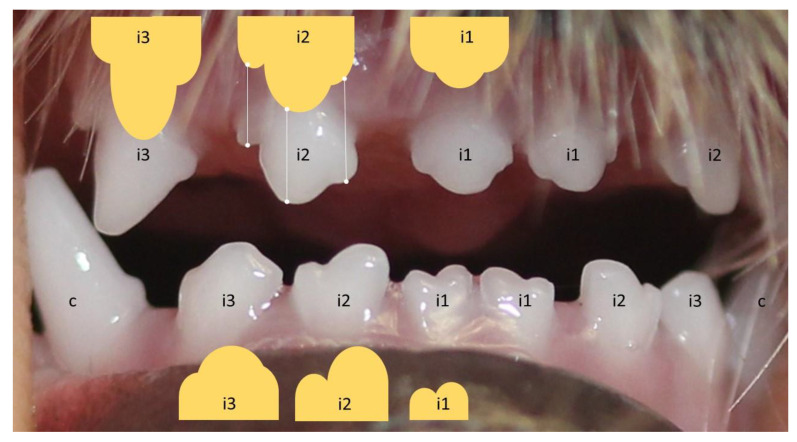
Special shaped incisors of a three-and-a-half-month-old Yorkshire Terrier.

For tooth shape and size, the 3.5 month status was used as a reference (Figure 1).

The two puppies developed their teeth at exactly the same rate. All images presented are from one of the dogs.

## 3. Results

**Figure 2:**
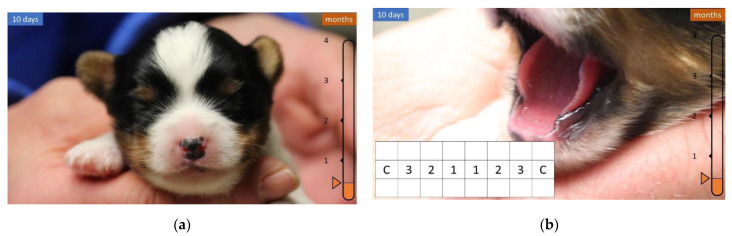
Development of the head and teeth in the first 10 days of life. (**a**) Near-spherical skull. (**b**) There are no teeth.

**Figure 3:**
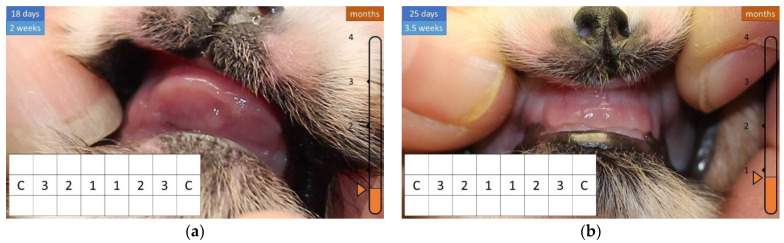
Development of the teeth in the first 18 and 25 days of life. (**a**) There are no teeth. (**b**) There are no teeth.

During the first few weeks of life, the head is shaped similar to a sphere and has no teeth (Figure 2 and Figure 3).

**Figure 4:**
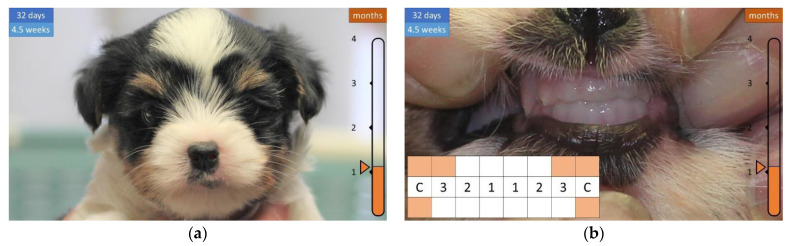
Development of the coat and teeth in the first 32 days of life. (**a**) Longer coat. (**b**) Appearance of the first teeth.

**Figure 5:**
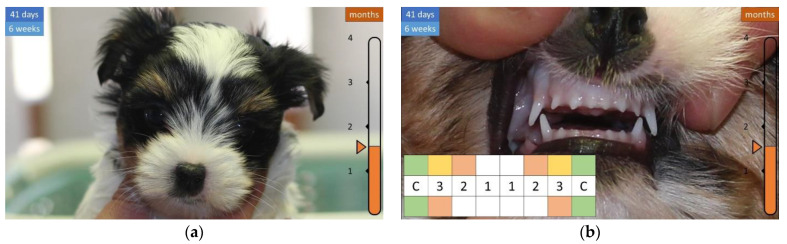
Development of the coat and teeth in a 6-week-old puppy. (**a**) Even longer coat. (**b**) Visible canines and new incisors.

At four and a half weeks of age, the coat is longer and the canines (c) and the adjacent incisors (i3) on the top (Figure 4) are erupting. At six weeks of age, the coat is even longer, the canine tooth (c) becomes significantly longer, and the second incisor (i2) emerges in the upper dentition (Figure 5).

**Figure 6:**
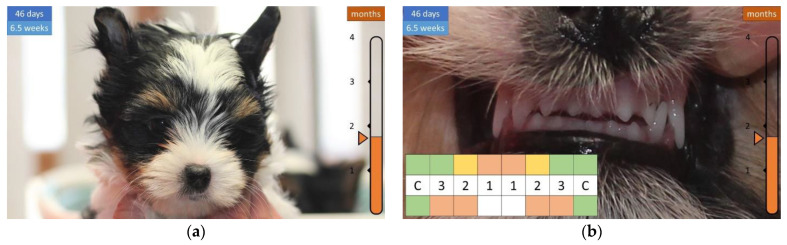
Development of the skull and teeth in a 6-week-old puppy. (**a**) Head shape is no longer spherical. (**b**) Upper dentition completed.

After a few days, the upper first incisor (i1) is also visible (Figure 6).

**Figure 7:**
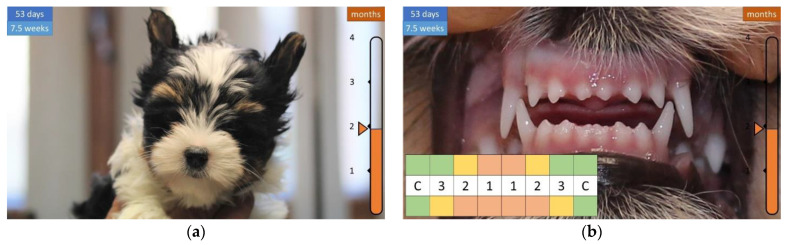
Development of the skull and teeth in a 7.5-week-old puppy. (**a**) An elongated face begins to form. (**b**) All teeth are visible.

**Figure 8:**
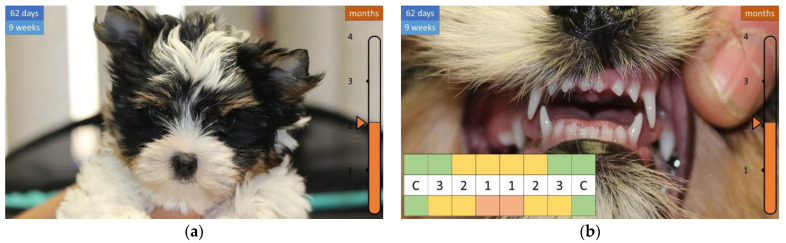
Development of the skull and teeth in a 2-month-old puppy. (**a**) More articulated head. (**b**) Most of the incisors are clearly visible.

**Figure 9:**
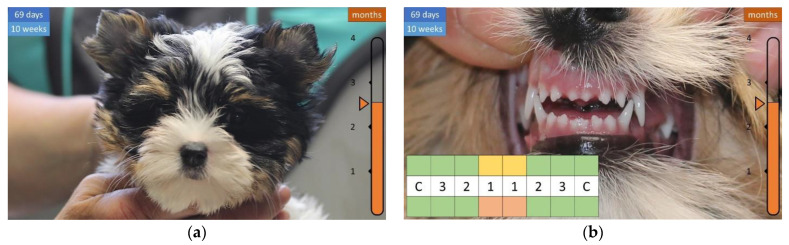
Development of the skull and teeth in a 10-week-old puppy. (**a**) Even more articulated head. (**b**) Teeth grew in a row except for the first incisors.

A week later, the face becomes longer and the lower first incisor (i1) appears (Figure 7), then the teeth continue to grow, and the shape of the head becomes more articulated, covered with a longer coat (Figure 8 and Figure 9).

**Figure 10:**
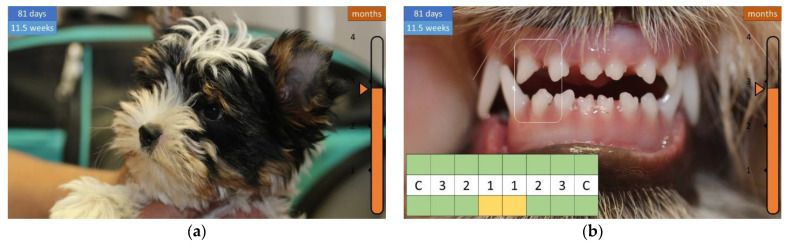
Development of the coat and teeth in a 11.5-week-old puppy. (**a**) Long coat (“tousled”). (**b**) The upper incisors are spaced apart.

At 2.5 months of age, the coat is long and the distance between the teeth is noticeable, more so for the upper dentition (Figure 10).

**Figure 11:**
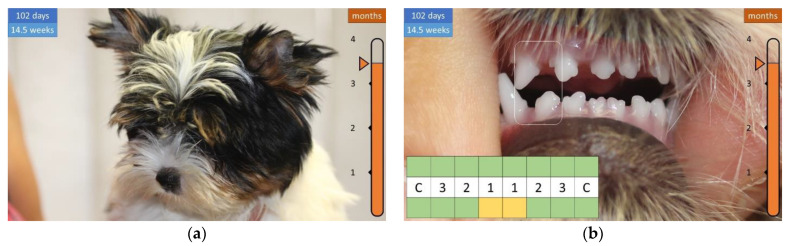
Development of the coat and teeth in a 14.5-week-old puppy. (**a**) Long coat that can be combed. (**b**) Distance between the lower teeth.

By 3 months of age, the coat is long enough to be combed, and the widening gap between the lower teeth can be observed in the bottom dentition, which corresponds with the upper incisors to about half the width of a tooth (Figure 11).

**Figure 12:**
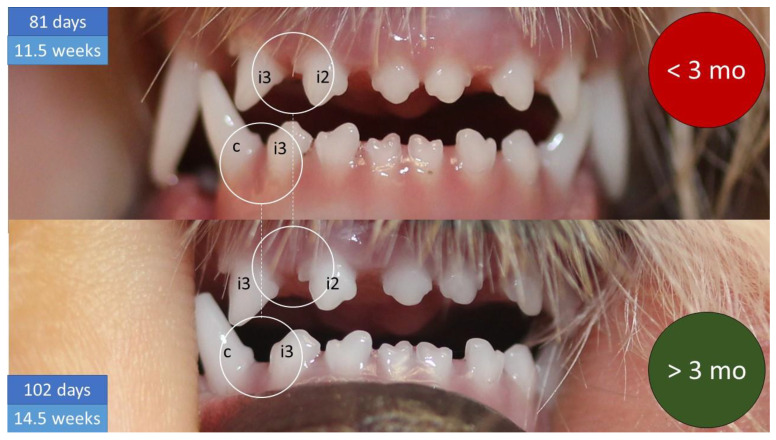
The gaps between the teeth of dogs under and over three months of age show a characteristic (about twofold) difference.

At 3.5 months of age, only the lower first incisors (i1) have not grown in a row. As the skull grows, the teeth become increasingly spaced apart. Under three months of age, the lower dental arch is barely visible, but after 3 months it is. In the upper dental arch the distance between the two extreme incisors (i2 and i3) is most prominent, and in the lower dental arch, the distance between the incisor (i3) and the canine (c) is the most prominent (Figure 12).

Gaps between the teeth of dogs under three months of age show a characteristic difference.

A specific condition is observed in the studied individuals for the period of time relevant in terms of the dog trade.

## 4. Discussion

A dog is born without teeth, then at a certain age the milk teeth appear (erupt), emerge, and grow to full size (grow in a row). The emergence and positioning are species specific [72]. The growing tooth mechanically excites the gums, causing them to bite and chew, which aids and accelerates its penetration through the gums [73]. As the body grows larger, the size of the teeth does not follow it, so they become increasingly spaced, and then the deciduous teeth fall out (exfoliate) and larger permanent teeth grow, of a size appropriate to the adult skull [74]. In humans, it is accepted that teeth only appear when there is sufficient space for them, and that tooth germs migrate to the desired location as the cranial bones grow, as a result of bone resorption and new bone formation [75]. For new teeth to erupt, the bones that hold the teeth need to grow properly [76]. The displacement of tooth germs and erupted teeth is aided by the increased local rate of bone formation, which even in adult dogs is three times that of the femur in the case of the jawbone and five times that in the case of the mandible [77]. Within the mandibula, the bone formation in the tooth sockets is 3–5 times faster than in the cortical bone [78]. In puppies, the rate of bone formation in the cortical bone of the jawbone is initially lower than in the femur, but slows down as age progresses, while the rate of bone formation in the jawbone and mandible remains high [79].

The milk incisors (i) and canines (c), which can be easily examined by viewing, appear in the first months of life. For veterinary practitioners, the dental characterization of the second to fourth months is not included in the literature [80]. One of the most prestigious professional bodies [81] in veterinary dentistry does not describe the emergence of milk teeth; instead, it refers in its nomenclature to a book on an anatomy nomenclature, which does not include dental information for the first few months [25]. The World Small Animal Veterinary Association’s [82] Global Dental Guidelines [83] contain detailed anatomical descriptions, but data on dentition are missing. The American Animal Hospital Association’s (AAHA) guidelines [84,85] for the dental examination and care of dogs and cats do not include the characteristics of dentition; the appearance table of deciduous teeth [86] relates to the period between 3 and 6 weeks of age, with a spread of several weeks for each tooth, and then with a spread of 1 to 2 months for permanent teeth after 4 months of age. In its guidelines describing the life stages of dogs [87], the AAHA discusses the age characteristics of dogs from two weeks of age, but only discusses teeth as a potential health problem. The age of three months cannot be established by using veterinary bulletins [88,89], nor even in the related papers of dental clinics [90], or it is not mentioned at all [91,92,93]. A world-renowned pet food industry player also provides information on possible age determination from teeth [94], but in stages overarching several months.

Determining age by simple means would have great advantages, but the method to determine age by dentition alone is uncertain as far as we currently know. It is possible that, similarly to the significant differences in ossification between species [75], tooth development may differ between individuals to such an extent that age estimation from teeth is too imprecise. There is little specific literature on any particular breed and even then, there may be considerations that affect the assessment and the final conclusion. In addition to hereditary traits, the time of emergence of human deciduous teeth depends on the composition of the diet [95,96,97,98], partly also on the texture of food [99], and therefore the results of the study on the teeth growth of the 107 beagles with the largest number of one breed may have been influenced [71] by the fact that the animals were fed twice daily until 4 months of age and only once thereafter. Today, puppies are fed three times a day up to six months of age in the most age-appropriate formulation and with a feed pellet size that takes into account the size of the breed.

In small dogs, dentition may be delayed [48], which is consistent with our observation that, in the small breed dogs we studied, the change in incisors to permanent teeth occurred much later than would have been expected based on previous experience [66]. Silver [47] described all incisors as erupted at the age of 4 to 6 weeks, whereas in the dogs we studied, mandibular i1 did not appear until the age of 6.5 weeks, only a week later. In the brachycephalic breeds, some teeth are more frequently unerupted than average [99], and although this abnormal cranial shape is not typical of the Yorkshire Terrier, it may have occurred in our case. The position of the growing teeth relative to each other varied from week to week, probably due to the non-uniform growth of the teeth [100].

## 5. Conclusions

The development of the two puppies’ dentition occurred at the same rate and was consistent with our decades of veterinary experience. The main value of the observations is the proven affiliation to a breed that is popular in the dog trade, the certainty of age and the pictures showing the progression of the teeth in relation to each other. Nevertheless, the results need to be extensively validated before any general conclusions are drawn, as only two dogs of one breed were involved in the study and several factors can be involved in the dentition, including individual differences and variability among breeds, especially if airorhynchy and klinorhynchy skulls are considered.

## Data Availability

The data presented in this study are available on request from the corresponding author without undue reservation.
